# Event and Comment

**Published:** 1914-10-15

**Authors:** 


### DENTAL REGISTER.

Vol. LXVIII. October 15, 1914. No. 10

### EVENT AND COMMENT.

THE SIXTH INTERNATIONAL DENTAL CONGRESS. This Congress was called to meet in London, August 4th, 1914, and in accordance therewith the opening session was held in Central Hall, Westminster. There was a good sized audience present when President J. Howard Mummery made his opening address. Responses to the address of welcome by Dr. W. B. Paterson, of London, were made by delegates from all the countries which were not involved in the great European war at the time. Owing to the general excitement incident to the opening of the war of England with Germany almost all the social functions that had been planned by the city were abandoned. In the afternoon of the first day a second general session was held, and Dr. E. C. Kirk, of Philadelphia, read a paper on "The Tendencies in Dental Education," and Dr. W. Guy, of Edinburg, read a paper on Narcosis. The next day was occupied with Section meetings and exhibits or rather with attempts to hold such meetings. Owing to the distractions which the war caused it did not seem possible for those who had charge of the Congress to hold the attendance, or to present such of the programme as was provided in an orderly way. The visiting delegates did not know what was to be done or where the meetings were to be held, as many changes in the programme were made without proper announcement, and as a consequence most of the foreign delegates spent most of their time making arrangements to get back home, or in endeavoring to get their checks cashed so that they could have the necessary money to take care of themselves. None of the clinics were given and the exhibits which had been set up in a hall that the government took possession of for war purposes, had to be hurriedly repacked and removed. The confusion also made it impracticable for the English dentist to extend any hospitalities to their guests. A number of the American delegates were hospitably cared for by Dr. J. Leon Williams and other American dentists in London. There were no delegates present from France, Germany, Austria or Russia, and only one or two from Italy. Taken altogether the Congress was a failure from all view points and no doubt it would have been postponed had any one dreamed that such unfavorable conditions could have been produced in so short a time. An effort was made to have the Congress adjourned for two years to meet at the same place, but this motion did not prevail and the matter is referred to the next meeting of the Federation Dentaire, which will take place at San Francisco next summer. Such of the addresses as were made and the reports will be published in the Britsh Dental Journals, and probably in book form for distribution to the members. It is unfortunate for all dental literature that this Congress failed so disastrously, as there is no doubt we should have presented at this time much of the best thought and work of dentists throughout the world, The next time we attempt to hold a Congress in Europe it might be well enough to see to it that the teeth of the European War Lords are all in good condition for a few months previous to meeting, so that their several digestions shall be normal and their tempers more courteously disposed.

EUROPEAN WAR. That the tremenduous war now going on in Europe may be detrimental to advancement of the science and art of dentistry there can be no question. During the last decade Germany especially has made tremendous advancement, especially in the sciences which are fundamental to the art of dentistry, and there is everywhere evidences that her dentists are beginning to apply the results of her scientific researches to the art of dentistry. It may be a long time before her older and conservative practitioners catch the Yankee spirit of invention and practice, but by nature the Teuton has the faculty of discernment and is not so bigoted as to prevent his advancement, especially where he knows he can better his own methods. This, Americans, who attended the Fourth International Dental Congress in Berlin, five years ago, could not fail to observe in the reproduction of practically every American dental instrument and equipment, even to the detail of design and decoration of our dental furniture. It is known that every man in Germany who is capable of going to war is today at the front, or on the way there, and the tales of the terrible loss of lives can not help but involve many lives that have been invaluable in the practice of dentistry, and the development of its science, or is engaged in the production of our valued medicines and instruments. When we think of the loss of all the lives that contribute to our profession in its many departments it brings this foreign war close home to us, and we begin to realize how dependent the world is on the welfare of all its peoples. It was no little thing that the Austrian Emperor pulled off when he sent his ultimatum to little Servia. It was the spark that has kindled a world blaze that will stop the whole worlds progress for many generations. Our newspapers have been speculating as to the advantages that were bound to come to America because of this war, but we have as yet experienced none of their prognastications; in fact we are in many ways experiencing discomforts and even financial loss as a consequence of the war. If this is so at this early date what are we to anticipate should this war be as prolonged and disastrous in men and other resources as it promises to be. We can most sincerely hope that the fortunes of war may be so decisive on one side or the other as to bring a speedy termination to the disastrous conflict.

ORAL HYGIENE'S NEW EDITOR. We are greatly pleased to welcome again to the editorial fraternity Dr. William W. Belcher, as editor of Oral Hygiene. We know of no one so capable and admirably equipped to succeed our lamented confrere, Dr. G. E. Hunt. He is full of knowledge as well as of the spirit which has made this journal the chief exponent of the public movement for better oral health. His wit and great heart will enable him to carry forward with honest purpose the great work which his predecessor had so much faith in, that he had determined to give the rest of his life to its advancement. We trust that Dr. Belcher may have the strength of body and mind to enlist the profession in behalf of this great work and that he shall see in his day the fulfillment of his predecessors dream, the abolishment of dental caries as a young peoples disease.

